# Laparoscopic Bilateral Adrenalectomy in a Young Female Patient with Recurrent Cushing's Disease

**DOI:** 10.1155/2021/6632436

**Published:** 2021-02-12

**Authors:** W. G. P. Kanchana, P. A. D. M. Kumarathunga, Gajawathana Shakthilingham, Charles Antonypillai, Sonali Gunatilake, D. D. Karunasagara, T. Jayasingharachchi, V. Pinto, K. B. Galketiya

**Affiliations:** ^1^Department of Surgery, Teaching Hospital Peradeniya, Kandy, Sri Lanka; ^2^Diabetes and Endocrine Clinic, National Hospital Kandy, Kandy, Sri Lanka; ^3^Department of Anaesthesiology, Teaching Hospital Peradeniya, Kandy, Sri Lanka

## Abstract

**Introduction:**

Synchronous bilateral adrenalectomy is undertaken less often due to numerous perioperative challenges and rare circumstances of patients needing this procedure. Bilateral adrenalectomy is an important second-line option for patients with persistent or recurrent hypercortisolism following transsphenoidal surgery for Cushing's disease. Here, we present a challenging case of synchronous laparoscopic bilateral adrenalectomy for a young female patient with recurrent Cushing's disease and fertility wishes. *Case Presentation*. A 21-year-old recently married patient who was diagnosed with Cushing's disease with a pituitary microadenoma had undergone two attempts of transsphenoidal excision of the pituitary tumour. Follow-up evaluation showed an unresectable residual tumour with invasion of the intracavernous part of the left internal carotid artery. As the patient had the hypothalamic-pituitary-ovarian axis intact with strong fertility wishes, she was offered bilateral adrenalectomy instead of radiotherapy. She was prepared for the surgery with close perioperative support from the endocrinology and anaesthesia teams. Intravenous hydrocortisone infusion was started at the induction of anaesthesia. Transperitoneal approach was used with the patient positioned in left and right lateral positions for right and left glands, respectively. A meticulous surgical technique was used for the identification of adrenal veins to clip them before division followed by handling of the glands. The patient had minimal haemodynamic disturbances during surgery. Intraoperative blood loss was less than 100 ml, and operative time was 220 minutes. She had a gradual recovery following postoperative respiratory distress due to basal atelectasis and consolidation. Cortisol levels were less than 20 nmol/L postoperatively, suggesting successful surgical intervention. Two months after surgery, she continued on maintenance therapy of oral hydrocortisone and fludrocortisone and was encouraged to go ahead with pregnancy.

**Conclusion:**

Although bilateral adrenalectomy is considered a high-risk procedure, these risks can be mitigated and performed safely while maintaining close multidisciplinary perioperative support.

## 1. Introduction

Synchronous bilateral adrenalectomy is undertaken less often due to numerous perioperative challenges and rare circumstances of patients needing this procedure [1]. Bilateral adrenalectomy is an important second-line option for patients with persistent or recurrent hypercortisolism following transsphenoidal surgery for Cushing's disease [2]. Other indications for bilateral adrenalectomy include ectopic ACTH-dependent Cushing's syndrome, adrenocortical hyperplasia, bilateral adrenal adenomas, and bilateral pheochromocytomas [3]. A majority of bilateral adrenalectomies are being done on Cushing's disease patients as seen in several case series [1, 3, 4].

Cushing's disease causes ACTH- (adrenocorticotropic hormone-) dependent rise in cortisol levels due to pituitary macro- or microadenoma. First-line therapy is transsphenoidal excision of the pituitary tumour. Persistent or recurrent Cushing's disease after transsphenoidal excision is often treated medically or by radiotherapy when the residual pituitary tumour is unresectable. Patients with failed medical therapy and fertility wishes which preclude radiotherapy are selected for bilateral adrenalectomy which aims to halt cortisol production and its effects [5].

Here, we present a challenging case of synchronous laparoscopic bilateral adrenalectomy for a young female patient with recurrent Cushing's disease and fertility wishes.

## 2. Case Presentation

A 21-year-old recently married female patient was evaluated for weight gain, oligomenorrhoea, and hirsutism which progressed over a duration of 2 years. She had a typical Cushingoid appearance and elevated blood pressure on examination. Her visual field assessment was normal. Overnight dexamethasone suppression test (ODST) and low-dose dexamethasone suppression test (LDST) failed to suppress the HPA (hypothalamic-pituitary-adrenal) axis confirming the diagnosis of Cushing's syndrome (CS). Subsequent evaluation revealed elevated ACTH levels and a 9.6 mm × 6.7 mm nonenhancing lesion in the pituitary fossa (pituitary microadenoma) leading to a diagnosis of Cushing's disease (CD).

**Figure 1:**
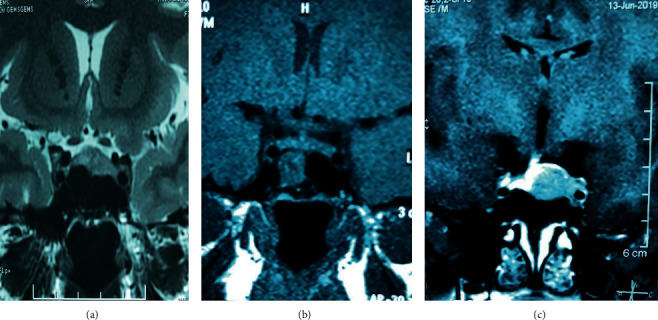
(a) 9.6 mm × 6.7 mm nonenhancing lesion in the pituitary fossa, (b) postsurgical changes with no evidence of adenoma, and (c) a 1.3 × 1.2 × 1.1 cm recurrent lesion in the left pituitary with left cavernous sinus invasion.

She underwent transsphenoidal excision of the pituitary microadenoma in May 2018, which resulted in remission. Histology was suggestive of a pituitary lesion with strong cytoplasmic positivity to ACTH. Follow-up revealed recovery of HPA axis within 6 months to preoperative values. Re-evaluation at 1 year revealed nonsupressed ODST and LDST with MRI evidence of recurrent tumour in the pituitary fossa with left-side cavernous sinus invasion confirming recurrent Cushing's disease (Figure 1).

She underwent repeated transsphenoidal excision of the pituitary tumour in August 2019. Histology was consistent with a pituitary adenoma with strong cytoplasmic staining for ACTH. No malignant features were seen in the histology, and the Ki-67 index was 8 to 10%. The immediate postoperative evaluation revealed persistently high ACTH and cortisol levels suggestive of persistent Cushing's disease. MRI showed residual tumour measuring 9 × 10 × 12 mm in size after 4 months from surgery with invasion of the intracavernous part of the left internal carotid artery.

At this point, with an unresectable residual pituitary tumour and medical management failing to control hypercortisolism, she was left with either radiotherapy or bilateral adrenalectomy for control of hypercortisolism. Since she had the hypothalamic-pituitary-ovarian axis intact with strong fertility wishes, she was offered bilateral adrenalectomy. Her preoperative ultrasound scan showed prominent bilateral adrenal glands.

She was prepared for surgery with close perioperative support from the endocrinology and anaesthesia teams. Particular attention was given to pulmonary prehabilitation, blood pressure, and blood sugar control. Intravenous hydrocortisone was initiated at the induction of anaesthesia and continued as an infusion.

She was initially operated on the left lateral position for the right adrenal gland. We employed the Veress needle technique with 5-port entry. The liver was retracted to expose the hepatorenal pouch. Blunt and sharp dissection continued to expose the inferior vena cava (IVC) and the upper pole of the right kidney. The right adrenal gland was identified. Dissection continued in the right side of the IVC and behind the IVC, separating the right adrenal gland from the IVC. The adrenal vein was identified above the level of the renal vein and clipped before division. The right adrenal gland was dissected off from the upper pole of the kidney and perinephric fat using ultrasonic shears and by bipolar diathermy. The specimen was delivered in a bag. Port sites were closed with a drain at the hepatorenal pouch.

**Figure 2:**
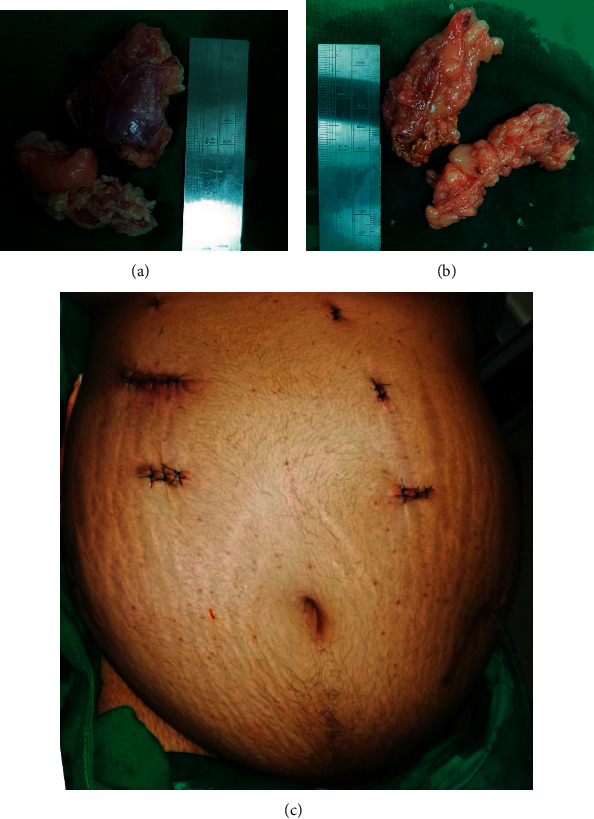
(a) Right adrenal gland with perinephric fat. (b) Left adrenal gland with perinephric fat. (c) Port placement and surgical wounds.

The patient was placed in the right lateral position for the left side procedure. The large pendulous abdomen of the patient resulted in all right-side ports being well away from the left surgical site. Thus, none of the ports used for the right side could be utilized for left side surgery (Figure 2). Veress needle technique with 5-port entry was employed. Splenic flexure was brought down by division of gastrocolic omentum and splenocolic attachments. Descending colon mobilized medially to expose the left kidney. The hilum and upper pole of the left kidney was exposed. The left renal vein, tail of the pancreas, and splenic hilum was exposed and identified using blunt and sharp dissection. The left adrenal vein originating from the left renal vein was identified and clipped before division. The left adrenal gland with surrounding perinephric fat was dissected with ultrasonic shears and bipolar diathermy. The specimen was retried in a bag. Port sites were closed with a drain at the left paracolic gutter. Figure 2 shows the large pendulous abdomen with closed port sites. The patient had minimal haemodynamic disturbances during surgery. Intraoperative blood loss was less than 100 ml, and operative time was 220 minutes.

The patient had a smooth recovery initially and was sent to ward from the intensive care unit (ICU). She developed respiratory distress and low oxygenation on the 2^nd^ postoperative day. Pulmonary embolism was excluded with a CT pulmonary angiogram. CT showed basal atelectasis and consolidation which was treated with CPAP (continuous positive airway pressure) mask, high-flow oxygen therapy, and antibiotics. Intravenous hydrocortisone infusion was continued to support the stress response to possible chest infection. She had a gradual recovery since then and was discharged on the 10^th^ postoperative day on oral hydrocortisone and fludrocortisone therapy.

Cortisol levels were performed on day 14 from surgery after withholding oral steroid therapy for 12 hours. Cortisol levels were less than 20 nmol/L suggestive of successful surgical intervention. Histology report showed bilateral hyperplastic adrenal glands. Two months after surgery, she continued on maintenance therapy of oral hydrocortisone and fludrocortisone and was encouraged to go ahead with pregnancy with close follow-up for the development of Nelson's syndrome.

## 3. Discussion

Since the first laparoscopic adrenalectomy was performed in 1992 by Gagner et al., laparoscopic adrenalectomy gained increasing popularity and is considered the gold standard in modern day surgical practice as the relatively small size of the adrenal gland does not justify a large surgical wound [6]. Laparoscopic approach is advantageous in Cushingoid patients as the open approach could lead to difficult access and large surgical wounds which in turn could lead to poor pulmonary function, chest infections, and wound infections following surgery [1, 7]. Apart from wound-related complications, open bilateral adrenalectomy has shown to be associated with higher rates of intraoperative and postoperative bleeding and higher morbidity (40% vs. 13%), and mortality (5.6% vs. 2.4%) rates when compared to the laparoscopic approach [1]. The patient presented did not require narcotic analgesics from day one and was very comfortable on the following day after surgery. However, she became tachypnoeic by postoperative day two and required readmission to ICU.

Bilateral adrenalectomy presents numerous perioperative and intraoperative challenges. Preoperative control of comorbidities is of great importance as uncontrolled diabetes or hypertension could further complicate perioperative care. Preoperative medical therapy may be indicated in patients with high mean cortisol burden. Intraoperative complications including bleeding, need for conversion to open surgery, and haemodynamic disturbances due to excessive handling of the gland can be avoided when meticulous and thorough surgical techniques are used [3, 6]. Our patient was stable throughout the procedure.

Commonly employed technique in adrenal surgery is the laparoscopic transperitoneal approach [8]. This allows visualization of other intraabdominal viscera, thus achieveing better spatial orientation and control when compared with the retroperitoneoscopic approach. The retroperitoneoscopic approach could be helpful in avoiding adhesions in patients with past abdominal surgeries as well as bilateral cases. Even though studies evaluating unilateral adrenal surgery using these two techniques have demonstrated no additional benefits for the retroperitoneoscopic approach, some authors have suggested benefits in bilateral surgeries as the prone position can allow access to both surgical sites avoiding the need for repositioning of the patient [1, 8]. We used the transperitoneal approach which is the standard technique in our unit.

Adrenal crisis remains the most feared complication after bilateral adrenalectomy and requires intravenous hydrocortisone therapy until the patient can tolerate oral hydrocortisone therapy. Some authors have reported infectious and thromboembolic complication rates as high as 40% and 13%, respectively [1]. Thus, mechanical and pharmacological thromboprophylaxis is warranted until the patient is fully mobilized. Our patient developed postoperative respiratory distress for which timely investigations and interventions were instituted, leading to satisfactory patient recovery.

Patients undergoing bilateral adrenalectomies need to be assessed for the development of Nelson's syndrome as the loss of feedback inhibition on the pituitary gland can lead to flare up of the tumour. Thus, assessment of ACTH levels and pituitary fossa MRI is necessary in the follow-up [5].

## 4. Conclusion

Although bilateral adrenalectomy is considered a high-risk procedure, these risks can be mitigated and performed safely while maintaining close multidisciplinary perioperative support.

## Data Availability

All data regarding the case are available upon request.

## Additional Points

*Patient Perspective*. “I was worried about going for radiotherapy to treat the residual tumour as doctors explained to me that I could have problems in conceiving. This was especially sensitive issue to me as I was married for 3 years and awaiting completion of my treatments to have children. Doctors explained that removing both adrenal glands is a procedure that should not be taken lightly as it might have complications. But having discussed with my husband, I took the decision to go ahead with this surgery with the hope of having children one day. At the end I am doing well following surgery and I am happy that I took this decision.”
